# Efficient Mapping and Tracking the Properties of Micromechanical Resonators Using Phase-Lock Loops with Closely-Spaced Frequencies

**DOI:** 10.3390/mi17020213

**Published:** 2026-02-05

**Authors:** Agnes Zinth, Samer Houri, Menno Poot

**Affiliations:** 1 Department of Physics, TUM School of Natural Sciences, Technical University of Munich, D-85748 Garching, Germany; 2 Department of Electrical Engineering, TUM School of Computation, Information and Technology, Technical University of Munich, D-85748 Garching, Germany; 3 Munich Center for Quantum Science and Technology (MCQST), D-80799 Munich, Germany; 4 IMEC—Interuniversity Microelectronics Centre, Kapeldreef 75, 3001 Leuven, Belgium; 5 Institute for Advanced Study, Technical University of Munich, D-85748 Garching, Germany

**Keywords:** MEMS and NEMS, phase-locked loop, membrane, silicon nitride, mode mapping, tracking

## Abstract

Studying the dynamical behavior of micro- and nano-mechanical systems (MEMSs and NEMSs) is essential in various fields from nonlinear dynamics to quantum technologies. Hence, it is important to precisely monitor the mechanical properties of MEMS and NEMS devices. In this work, we show how to track and spatially map various properties of a mechanical resonator, such as frequency shift, linewidth, and nonlinearity, by appropriately selecting three closely spaced drive frequencies and using phase-locked loops. This technique tracks changes in the system quickly and efficiently, without the need for repeated frequency sweeps of the oscillator response, simply by employing three phase-locked tones.

## 1. Introduction

Micromechanical systems have become a vital component of various technologies, including sensing. This ranges from gas detection to quantum applications [1,2,3,4,5], and communication systems [6], biomedical instrumentation [7], even to applications such as cancer diagnosis [8]. The performance and reliability of these devices are strongly influenced by their dynamic characteristics, particularly the frequency response, which determines key parameters such as resonance frequency, damping rate, and nonlinearities. Precise frequency-response characterization is therefore essential for monitoring these, but also for the understanding systems’ dynamics and ensuring stable operation under varying conditions. Traditional approaches—like recording linewidth maps of 2D material resonators [9], monitoring magnetic and electronic phase transitions [10] or optimizing a fabrication process, e.g., the influence of annealing on the quality factor [11,12,13]—to characterize mechanical resonators, typically involve sweeping the frequency of the driving force over the resonance to measure the amplitude and phase response. While effective, frequency sweeps are time-consuming and prone to drift over time, which limits their ability to track rapid changes. To overcome these limitations, frequency tracking can be achieved through phase-locked loops (PLLs). This has, e.g., enabled continuous monitoring of resonance frequencies with high precision [5,14,15,16]. Similar to the multi-frequency AFM technique, where either the first two modes [17,18] or higher harmonics [19] are used, our technique also uses several frequencies to gain information about resonators.

Here, we present a method for efficiently mapping and simultaneously tracking multiple properties—specifically, linewidth, frequency, and nonlinearity—of a mechanical resonator. We select three closely spaced frequencies within the width of a resonance peak, then follow them with PLLs and determine the desired quantities from the PLL outputs. First, we cover the concept behind our technique and then present the experimental realization in a micromechanical membrane. We start by tracking the resonators’ properties locally during a temperature sweep, then extend to spatially mapping them. Lastly, the nonlinear regime will be explored.

## 2. Concept

**Figure 1:**
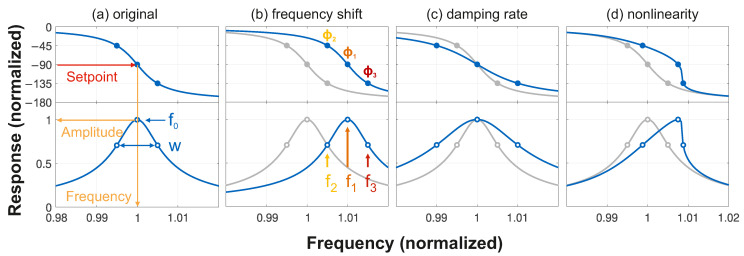
Illustration of the concept showing the frequency response (blue) of a driven harmonic oscillator with phase (top, in deg.) and magnitude (bottom) for every panel. (**a**) PLLs can be used to lock to specific phases (filled symbols), resulting in different driving frequencies and amplitudes (open symbols). A shift in resonance frequency (**b**), damping rate (**c**), and nonlinearity (**d**) all result in distinguishable shifts in $f_{i}$. The original response with $w/f_{0}=0.01$ and α=0 is shown in gray.

For tracking properties of a resonator, phase-locked loops (PLLs) are instrumental [14,20,21]. A PLL is locked to its setpoint as upon changes in phase, the frequency is adjusted accordingly, as illustrated in Figure 1a. The information can be in the frequency and/or in the amplitude. For example, we used the latter to record mode maps with high resolution, while remaining stable against external influences [22,23]. There, we chose one frequency per mode to map up to six modes of a membrane simultaneously, but here we use three frequencies within the peak of a *single* mode, see Figure 1. By choosing three setpoints $\phi_{1,2,3}$—indicated by the filled symbols—instead of one per mode, we gain access to properties beyond amplitude and resonance frequency. These are traditionally obtained by taking frequency scans with a network analyzer (NWA) [24], which can be very time consuming.

As we will show in this section, with well-chosen setpoints, not only the frequency shift, but also the damping rate, and even the nonlinearity of the system can be determined straightforwardly. Traditionally, a setpoint of $\phi =\phi_{1}=-90^{\circ }$, corresponding to the maximum amplitude, tracks the resonance frequency $f_{0}$ (Figure 1a). Here, we use two additional PLLs. For a shift in resonance frequency $f_{0}$, all three frequencies move in unison as depicted in Figure 1b. The frequency shift can thus be determined directly from $f_{1}$. Figure 1c shows an increase in damping. Now, the center frequency $f_{1}$ remains unchanged, whereas $f_{2}$ and $f_{3}$ move apart symmetrically. In addition to the usual setpoint of $\phi_{1}=-90^{\circ }$, we select the two setpoints $\phi_{2}=-45^{\circ }$ and $\phi_{3}=-135^{\circ }$ strategically, so that $f_{3}-f_{2}$ directly yields the second parameter, the linewidth *w*, as we will see now. When driving our resonator at low powers, it behaves as a linear harmonic oscillator. The amplitude is given by the magnitude |H(f)| of the frequency response function H(f), whereas its phase ϕ=∠H(f) quantifies the delay between the drive and resulting motion. For simplicity, we focus on the case where the linewidth *w* is much smaller than the natural frequency $f_{0}$ and $|f-f_{0}|\ll f_{0}$ so that we can apply the Lorentzian approximation.

(1) $$\begin{array}{c} H\left(f\right)=\frac{1}{{\left(2\pi \right)}^{2}\;m}\frac{1}{f_{0}^{2}-f^{2}+iwf}\approx \frac{1}{{\left(2\pi \right)}^{2}\;m}\frac{1}{-2f_{0}\left(f-f_{0}\right)+iw\;f_{0}}\\ =\frac{1}{2{\left(2\pi \right)}^{2}\;m\;f_{0}}\frac{f_{0}-f-\frac{iw}{2}}{{\left(f_{0}-f\right)}^{2}+{\left(\frac{w}{2}\right)}^{2}}. \end{array}$$

Note that, w=γ/2π, where γ is the damping rate. Further, *m* is the mass of the resonator. The phase ∠H(f) under the Lorentzian approximation is given by the following:

(2) $$\tan\left(\phi \right)=-\frac{\frac{w}{2}}{f-f_{0}}\to \phi =-\frac{\pi }{2}-\arctan(\frac{f-f_{0}}{\frac{w}{2}}).$$

As the PLL phase and frequency are the measured quantities in our experiment, we want to utilize them to determine the linewidth and frequency. Rearranging the equation to calculate the linewidth *w* gives the following:

(3) $$w=2\;\left(f-f_{0}\right)\;\tan\left(-\phi \right)$$

or

(4) $$f=f_{0}+\frac{1}{2}w\cot\left(\phi \right).$$

Choosing three setpoints also gives access to three phases, which we can use to determine our resonators’ properties. Specifically, for $\phi_{1}=-90^{\circ }$, $f_{1}=f_{0}$. For $\phi_{2}=-45^{\circ }$, $f_{2}=f_{0}-w/2$ and for $\phi_{3}=-135^{\circ }$, $f_{3}=f_{0}+w/2$ so that $f_{0}=f_{1}$ and $w=f_{3}-f_{2}$.

Still, with three frequencies, an additional property can be determined. Panel (d) shows that for increasing nonlinearity—as quantified by the Duffing parameter α [25]—the phase of the response steepens on one side and flattens on the other. Thus, the equality between $f_{3}-f_{1}$ and $f_{2}-f_{1}$ no longer persists; this can be used to determine α. However, operation in the nonlinear regime is potentially more complex than this simple picture, as we will see later. In the following, we first focus on the experimental realization in the linear regime and then return to investigate the nonlinearity.

## 3. Results and Discussion

**Figure 2:**
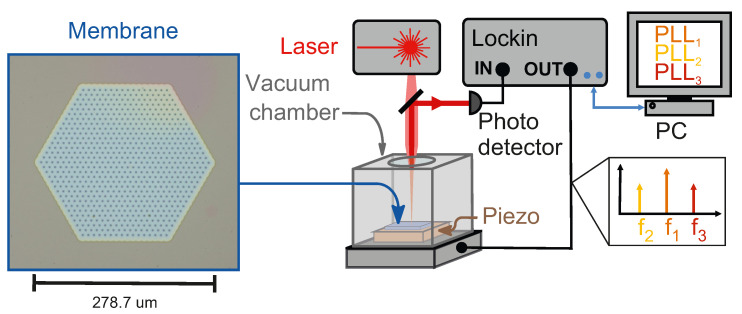
Schematic of the setup with a lock-in amplifier and digital implementation of the PLLs; for details, see Ref. [22]. The optical micrograph shows the actual membrane used in the experiments.

The resonator we use to demonstrate our technique is a hexagonal micromechanical membrane made from high-stress silicon nitride (SiN) [26], as further described in the Methods Section (Appendix A). The sample is mounted on a piezo and excited with a lock-in amplifier, as shown in Figure 2. This particular membrane has a number of (unintentional) features, like thickness variations or particles on the devices, that are instrumental in showing the applicability of our technique in real-life situations, as discussed below.

**Figure 3:**
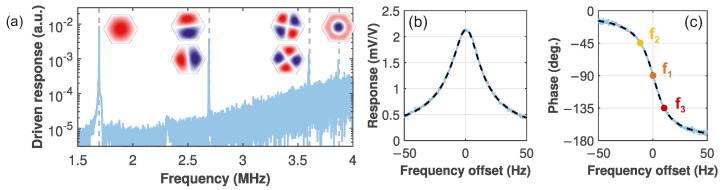
(**a**) Driven response of a hexagonal SiN membrane with resonances near the simulated eigenfrequencies (dashed lines). The corresponding mode shapes are shown as insets. (**b**) Magnitude and (**c**) phase of the fundamental mode. The setpoints are indicated as symbols.

Figure 3a shows the driven response of the membrane in vacuum. The different drum modes are visible and the insets show the simulated mode shapes of the first six modes. In the following, we focus on the fundamental mode near $f_{0}=1.69\;\mathrm{MHz}$. The zooms [(b),(c)] display an exemplary resonance that is fitted well by a harmonic oscillator response [Equation (1)] (dashed), yielding a linewidth of w=21.79 Hz.

### 3.1. Frequency and Linewidth Tracking During Temperature Sweep

**Figure 4:**
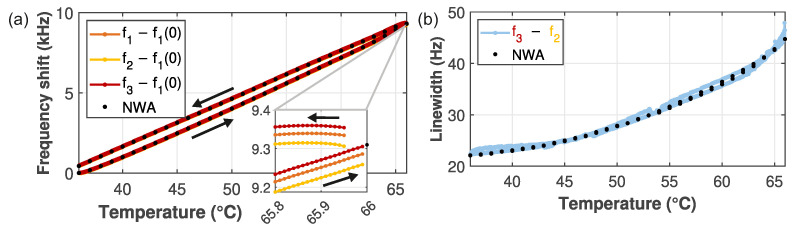
Tracking the (**a**) frequency shift and (**b**) linewidth during a large temperature ramp. Black data points indicate calibration NWA measurements. The inset in (**a**) is a zoom-in at the reversal point in the temperature curve, showing a small offset. The arrows indicate the sweep direction.

The first demonstration of our technique is the tracking of the resonator’s properties during a large temperature ramp. The temperature was swept up and down from 36 to 66 °C over the course of six hours with the PLLs tracking the changes. Figure 4a shows the frequency shifts and (b) the linewidth as a function of temperature. NWA calibrations were taken at fixed intervals (black dots), and these match the results from our PLL well. Overall, the resonance frequency shifts up by 10 kHz: The substrate expands more than the membrane, resulting in a higher resonance frequency. The frequency shift shows hysteretic behavior as the temperature of the sample mount changes, but the chip temperature lags behind. This is also the cause for the offset, visible in the inset: The last point of the upward sweep is recorded, and an NWA measurement is done (≈33s). During this time, the setpoint temperature already drops, but that of the chip still rises. Hence, afterwards the first point of the downward measurement starts at a slightly lower temperature but higher frequency.

The corresponding linewidth in Figure 4b has more than doubled. This is due to increased coupling between the membrane and substrate modes [27]. The determined values again nicely match the NWA measurements, but extracting the linewidth precisely from the NWA traces requires some tricks in the data processing. These and other technical details can be found in Appendix B. Still, the measurement time is far longer than for the PLL technique.

A closer look at the inset in Figure 4a reveals something unexpected: there is an asymmetry in the frequency differences. This is because the PLLs need time to follow rapid changes. The asymmetry of $f_{2}$ and $f_{3}$ with respect to $f_{1}$ arises due to the dependence of the PLL response on the shape of the phase response. This results in a different lag for the different setpoints for a continuous frequency shift. This effect is explained in more detail Appendix D. To compensate for this, we use an estimator in the post-processing of the data, taking into account the tracking error of the PLL. The three phases $\phi_{i}$ and corresponding frequencies $f_{i}$ form a linear system of equations that can be solved, giving more accurate estimations for $f_{0}$ and *w* as demonstrated in the next Section.

### 3.2. Estimator

Up to now, there has been no distinction between the setpoint of the PLL and the actual phase. From the experiment, we know the measured phase consists of the PLL setpoint $\phi_{i,\mathrm{setpoint}}$ and its error $e_{i}$:

(5) $$\phi_{i}=\phi_{i,\mathrm{setpoint}}+e_{i}.$$

The errors $e_{i}$ contain both a contribution due to the offset between the current frequency and the ideal one, as well as noise. The latter can be controlled via the bandwidth of the lock-in demodulator. The former may originate from, e.g., the lag of the PLL, but can be compensated for, as we will show now. With the three phases $\phi_{i}$, we can set up a linear equation system based on Equation (4), allowing us to determine the estimated linewidth $w_{\mathrm{est}}$ and frequency $f_{0,\mathrm{est}}$. This system of three linear equations and two unknowns is solved (as the least squares solution) by Matlab’s \-operator:

(6) $$\left[\begin{array}{c} f_{1}\\ f_{2}\\ f_{3} \end{array}\right]=\left[\begin{array}{cc} \cot\phi_{1} & 1\\ \cot\phi_{2} & 1\\ \cot\phi_{3} & 1 \end{array}\right]\left[\begin{array}{c} \frac{1}{2}w_{\mathrm{est}}\\ f_{0,\mathrm{est}} \end{array}\right]\to \left[\begin{array}{c} \frac{1}{2}w_{\mathrm{est}}\\ f_{0,\mathrm{est}} \end{array}\right]=\left[\begin{array}{cc} \cot\phi_{1} & 1\\ \cot\phi_{2} & 1\\ \cot\phi_{3} & 1 \end{array}\right]\setminus \left[\begin{array}{c} f_{1}\\ f_{2}\\ f_{3} \end{array}\right]$$

The result contains the estimated frequency $f_{0,\mathrm{est}}$ and linewidth $w_{\mathrm{est}}$. Note that, absent noise, Equation (6) gives the exact values independently of the PLLs being at their setpoints. Readout noise mainly affects $\phi_{i}$, resulting in deviations in the estimated parameters. The exact effect depends on the setpoints and PLL operations, but, in general, the more noise, the larger the deviations will be. As two parameters are sufficient to determine frequency and linewidth, the third can be used to determine an additional property, or simply for a consistency check.

**Figure 5:**
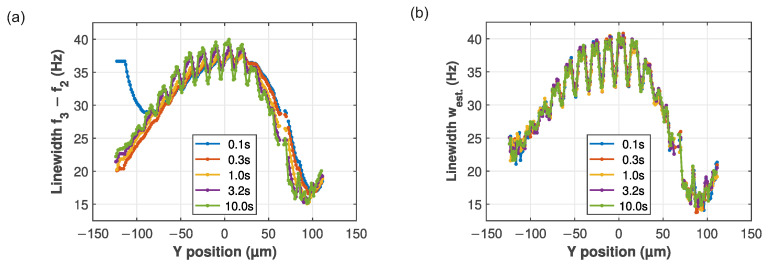
Comparison of the linewidth extraction methods for different wait times between steps: (**a**) $f_{3}-f_{2}$ and (**b**) the estimator $w_{\mathrm{est}}$. Feedback settings: $g_{\mathrm{FB}}=250\;V/V$ and $\theta_{\mathrm{FB}}=-90{}^{\circ}$.

To demonstrate the benefits of using the estimator, we compare both methods of extracting the linewidth in Figure 5. There, although the spatial dependence will be covered in detail in the next section, we already step over the membrane, recording the three PLLs for different waiting times between movements. Throughout the trace, sharp features from the membrane holes appear, which the PLL has to follow. If the waiting time is long enough, it will be able to adjust and $f_{3}-f_{2}=w$. However, for short waiting times, $f_{3}-f_{2}$ shows a long distance before settling, and no sharp features are seen. Increasing the wait to 10 s per point sharpens the features in the line trace, but recording a correct map would require long measurement times. Also, the ability to track rapid changes is lost when the PLL needs 10 s per point to follow the changes. The short waits are also shifted to the right, indicating a delay between the changing linewidth and the reaction of the PLL. In contrast, with $w_{\mathrm{est}}$, all traces coincide and show the sharp features, even for the shortest wait of 0.1 s. The estimator thus enables high-resolution mapping and tracking with short measurement times.

### 3.3. Mapping of Resonator Properties

From measuring at one position on the membrane, we extend to full spatial mapping of its properties. However, as neither systematic frequency shift nor linewidth change with position is expected in our membrane, we introduce these by implementing a feedback loop that feeds the measured displacement back with a controllable gain $g_{\mathrm{FB}}$ and phase shift $\theta_{\mathrm{FB}}$ [28,29] as detailed in Appendix A. A feedback force that is in phase with the displacement shifts the frequency, whereas feedback that is in phase with the velocity alters the damping, i.e., the linewidth *w* [30]. Since the total loop gain depends on $g_{\mathrm{FB}}$, as well as on the open-loop response (varying on the micrometer scale due to the release holes and slower due to the mode shape and readout), this results in both fine and large-scale features in the local frequency shift and damping rate.

**Figure 6:**
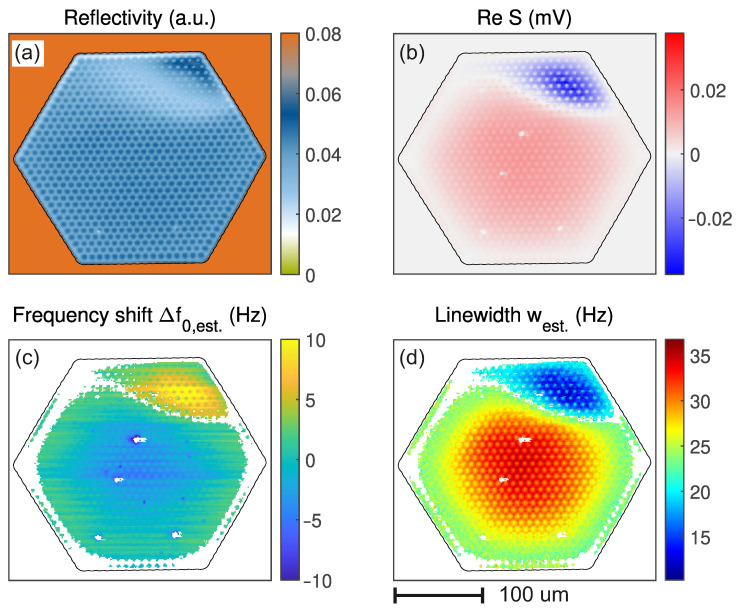
Maps in the presence of feedback ($g_{\mathrm{FB}}=250\;V/V$, $\theta_{\mathrm{FB}}=-45{}^{\circ}$). (**a**) reflected laser light, (**b**) signal amplitude, i.e., the product of the mode shape and transduction (see Ref. [22] for details on the meaning of ReS). (**c**) the frequency shift and (**d**) the estimated linewidth. The black line is a reflectivity contour that outlines the edge of the membrane. White in (**c**,**d**) are excluded regions where the signal is too low for the PLL to update the frequencies, or the error for the estimator is too large.

**Figure A5:**
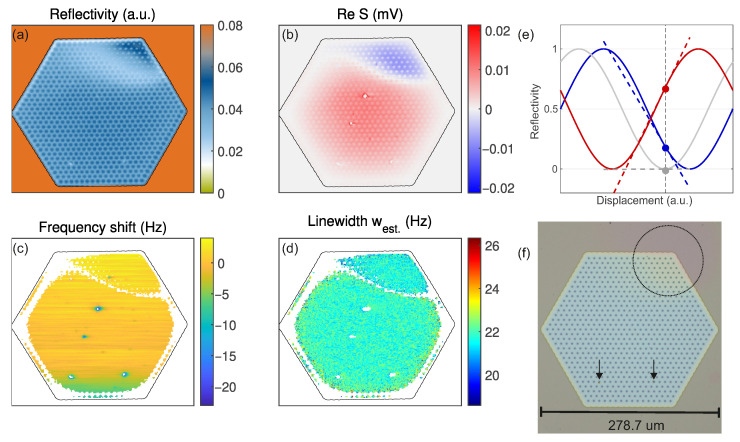
Maps without feedback (**a**) reflectivity, (**b**) amplitude, (**c**) the frequency shift, (**d**) estimated linewidth, (**e**) schematic reflectivity vs. displacement for different SiN thicknesses to explain the sign change in the transduction, (**f**) optical microscope picture indicating the dimension of the membrane and the spot in the upper right corner (circle). The arrows indicate particles on the membrane.

Figure 6 shows the spatial map of various properties in the presence of feedback: (a) shows the reflectivity and (b) the amplitude of PLL 1. The latter deviates from the expected mode shape [Figure 3a], as the membrane has a region with a different SiN thickness where the sign of the readout is reversed. This is explained in more detail in Appendix C Figure A5 [31]. Figure 6c shows the frequency shift; (d) the estimated linewidth. White spots in any of the maps indicate contamination on the membrane where the laser locally heats the membrane, shifting $f_{0}$ down sharply and reducing the signal. These points are either excluded due to low signal or large errors $e_{i}$. Yet, the PLL tracking remains robust to such real-world features, including sign reversal.

In the center of the membrane, the frequency shifts ≈10 Hz downward relative to the edge, and in the upper-right corner it shifts upward as the loop gain switches sign. Overall, a very fine structure is visible, and even the holes in the membrane can be resolved. A similar pattern occurs in the linewidth (d). There, the linewidth increases at the center of the membrane and decreases in the upper-right corner, again due to the applied feedback. Without it, the linewidth is constant at ≈22 Hz on the whole membrane, as shown in Figure A5. There, the same measurement without feedback is shown.

**Figure 7:**
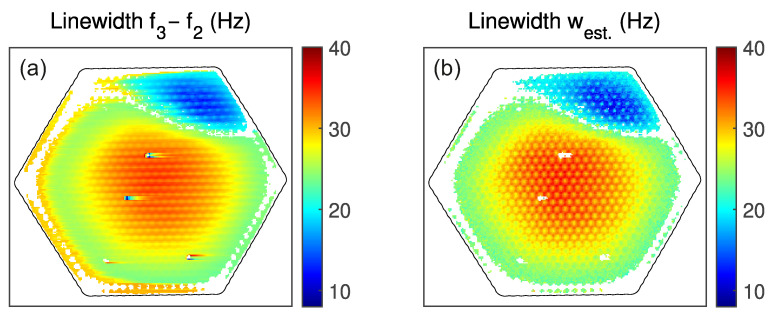
Comparison of the linewidth extraction from the same data using two methods. (**a**) $f_{3}-f_{2}$, (**b**) estimator $w_{\mathrm{est}}$. The measurement is taken by stepping left to right, then upward, line by line. Feedback settings: $g_{\mathrm{FB}}=250\;V/V$ and $\theta_{\mathrm{FB}}=-90{}^{\circ}$.

Also, for the maps, the estimator is applied during data post-processing, highlighting the methods’ benefits. Figure 7 compares the maps of the linewidth in the presence of feedback using the two methods on the same data. Both maps show similar magnitudes and trends, with the largest damping in the center and the smallest in the upper right corner. The map of $f_{3}-f_{2}$, also shows a larger linewidth at the lower and right edges. This is, however, because the PLLs need time to settle into the correct frequencies, and the errors are still large. Although the holes are visible, the picture appears rather blurred. In contrast, the map generated using the estimator $w_{\mathrm{est}}$ shows much finer features and does not exhibit artifacts such as unexpected high linewidths at the edges or at the spots where particles are located. Further $w_{\mathrm{est}}$ only needs a short time to settle after the particles, unlike $f_{3}-f_{2}$, where streaks are visible on the right side of the particles.

As each point only takes ≲2s (mainly limited by stage motion), mapping such fine structures in *w* and $f_{0}$ can be done much faster with our three-tone PLL than by recording a whole NWA trace for every single point over the whole membrane [22,32], which would take weeks to complete.

### 3.4. Operation in the Nonlinear Regime

**Figure 8:**
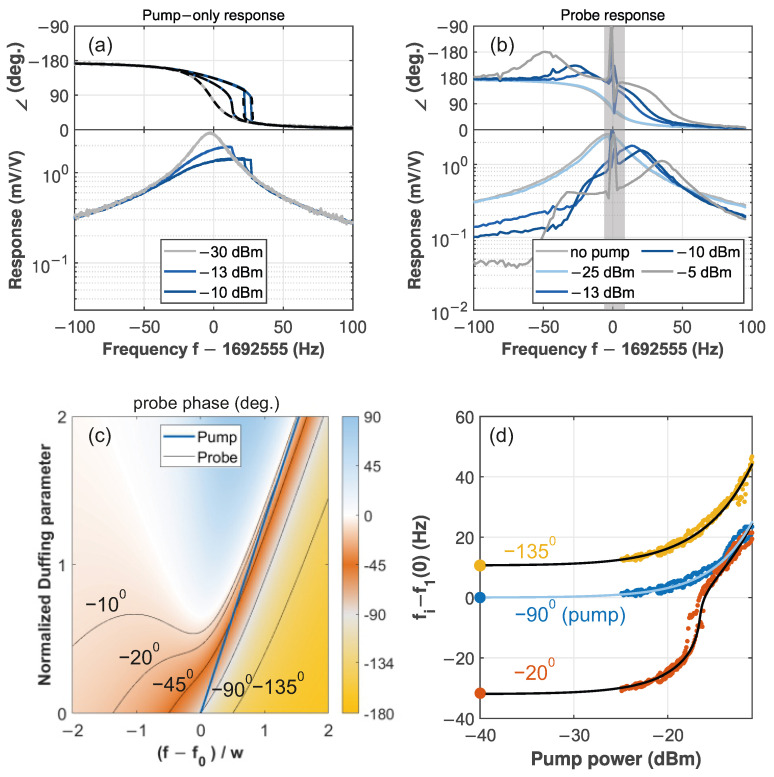
Operation in the nonlinear regime. (**a**) Driven responses for varying excitation power. The dashed lines show the fit of the phase. (**b**) Response of a weak (−30 dBm) probe for different pump powers. The pump at 1,692,555 Hz is grayed out. (**c**) Calculated probe response phase for different probe frequencies and strength of the nonlinearity. Contour lines at the indicated values are shown in black; the blue line shows the frequency of pump when locked to $-90^{\circ }$. (**d**) Evolution of the PLL frequencies for increasing pump strength. The pump is in blue and the two probes are gold and orange. For clarity, the markers at −40 dBm have been enlarged. The solid lines are the scaled contours from (**c**).

The final property that can be determined with our technique is the nonlinearity. Thus far, the membrane has been driven in the linear regime; increasing the driving allows access to the nonlinear response. In Figure 8a the driven response for different driving powers with only *one* tone (“pump”) is shown. When the drive increases, the resonance takes the shape of a Duffing response. Although the amplitude is affected by nonlinear readout, the phase—used by the PLLs—is not [26]. Its slope gets steeper with increasing excitation power, as was shown in Figure 1d so an asymmetry between $f_{3}-f_{1}$ and $f_{1}-f_{2}$ could be expected. Unfortunately, the nonlinearity makes the situation more complex: to apply our technique, we require three driving tones, but already adding a second, strong tone gives rise to very complex effects [33,34]. Hence, instead of equally strong tones for all PLLs, we use weak probes [35]. Figure 8b shows the responses of one probe for varying pump powers. Starting with a single relatively weak pump tone (−25 dBm) at a fixed frequency, the probe response is only slightly shifted compared to the case with no pump (gray). This one, in turn, closely matches the original linear response. Increasing the pump power leads to more complex probe responses. This is still captured by an analytical expression [36], which also shows that adding another weak probe can be done independently, see Figure A5. For frequencies higher than the pump, the measured amplitude and phase in Figure 8b look like a shifted linear response; the stronger the pump, the larger the shift. For lower frequencies, the response becomes more complex, with the phase developing a peak with positive values. Figure 8c shows a color plot of the calculated probe response phase versus nonlinearity induced by a $-90^{\circ }$-locked strong pump. Note that PLLs would follow the contour lines. Like the traces in (b), all setpoints shift towards higher frequencies. The pump at $-90^{\circ }$ is indicated in blue; the $-135^{\circ }$ line remains on the right side of the pump, hence we keep that setpoint for $\phi_{3}$. However, the previous $\phi_{2}=-45^{\circ }$ would cross the pump relatively quickly as the pump power increases. Hence, in the presence of small nonlinearities, PLL 2’s lock would already be lost since the frequencies approach each other. Therefore, a different setpoint has to be chosen. Indicated in Figure 8c are $-10^{\circ }$ and $-20^{\circ }$. The trend of the former is no longer monotonic and would therefore lead to loss of PLL lock when increasing or decreasing pump power. Contrarily, the $-20^{\circ }$ setpoint is farther from the pump than $-45^{\circ }$, while still being monotonous, and can therefore track higher pump powers.

The measurement with the adjusted values is shown in Figure 8d. The black lines indicate the scaled lines from (c). The different colors represent measured data points that match the theoretical curves well. In the linear regime, for small pump powers, the PLL frequencies evolve in unison. When increasing the pump, they shift upward as the response enters the Duffing regime. Above −20 dBm, a rather sharp transition is reached. There, the $-20^{\circ }$ setpoint approaches the pump frequency but still matches the calculations. In the experiment, there is a limit on how close two frequencies can get. More importantly, from the scaling of the contour lines, not only $f_{0}$ and w=22 Hz are obtained, but also the amount of nonlinearity. Specifically, we find a critical pump power of $P_{c}=-12.7\;\mathrm{dBm}$. This matches well with the value of −12.57 dBm that was determined independently from driven responses acquired at different network analyzer powers as discussed in Appendix E. Hence, not only the linear properties but also the nonlinearity of the strongly driven resonator can be determined using our method.

### 3.5. Discussion

In this final section, we discuss further possibilities of applying and extending the presented method. Until now, we demonstrated single-mode operation. The method can easily be extended to track two modes, as the lock-in we use has six demodulators. This is not a fundamental limitation; with a different measurement device, the number of modes can be increased even further. In general, the operation relies on the steadiness of the phase response. For modes that remain far in frequency, this is usually not an issue. Also, as long as ϕ(f) does not change, our method will also work for densely spaced modes. Only when nearly degenerate modes begin to interact does the phase response change, preventing reliable operation.

Currently, the available frequency range is limited by the piezo actuator. It has a resonance frequency of 21 MHz, after which the amplitude decreases. To expand the available range, one could switch to an electrostatic or an optical actuation scheme. Ultimately, the measurement is limited by the signal coming from the resonator, as the phase must be reliable enough to lock to. Further, there are different ways to implement the PLLs. One could, e.g., use dedicated hardware, enabling a faster operation and more deterministic timing. Specifically, using the real-time kit of the lock-in to implement the PLLs is a possibility to track sub-second changes with the current setup.

Finally, we emphasize that our technique is not limited to optomechanical readout of micromechanical membranes and can be applied to various systems. It is generic, i.e., independent of the optical readout and piezo actuation used in our case. The requirements for our method are solely the ability to perform a PLL measurement and a sufficient signal-to-noise ratio, so that the phase remains stable enough to remain locked throughout the measurement. We believe that the method can be straightforwardly applied to a wide variety of resonators, including, e.g., MEMS with capacitive readout and electrostatic actuation.

## 4. Conclusions

In conclusion, we demonstrate a fast and efficient method for tracking and mapping properties of a mechanical resonator, such as frequency shift, linewidth, and nonlinearity, using three strategically chosen driving frequencies instead of repeatedly recording frequency sweeps. This robust method allows us to investigate a range of unintentional features of our resonator, such as thickness variations, particles on the membrane, and temperature-dependent linewidth. Thus, it enables rapid characterization, spatial mapping, and real-time monitoring of the properties of changing systems.

## Figures and Tables

**Figure A1:**
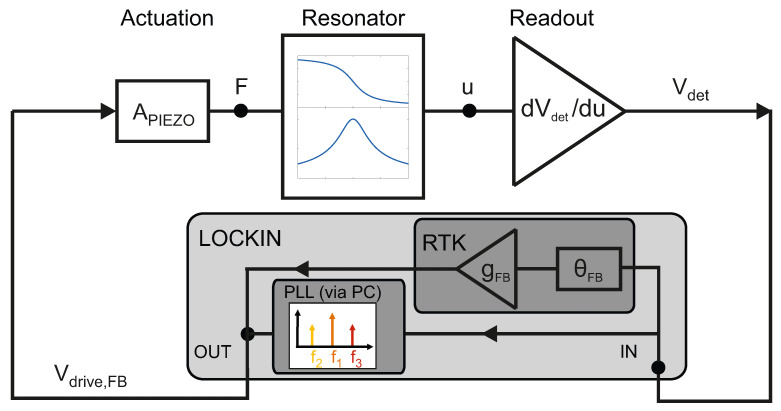
Sketch of the feedback part in the setup. The lock-in outputs the drive for the three PLLs, and a program is uploaded to the internal processor (RTK) to generate the feedback signal. The output actuates the piezo, resulting in the displacement of the membrane. The change in reflected power is detected by the photodetector. After amplification, this gives the signal $V_{\det}$.

**Figure A2:**
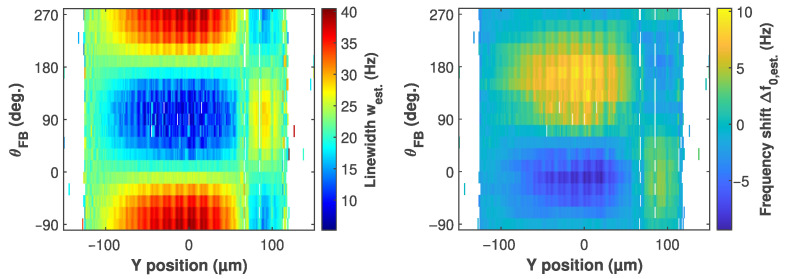
Color maps of the estimated linewidth (**left**) and frequency shift (**right**) as a function of the feedback phase $\theta_{\mathrm{FB}}$ while stepping over the membrane with a feedback gain of $g_{\mathrm{FB}}=250\;V/V$. Note how the feedback-induced shifts reverse sign when the transduction becomes negative beyond y≳70μm.

**Figure A3:**
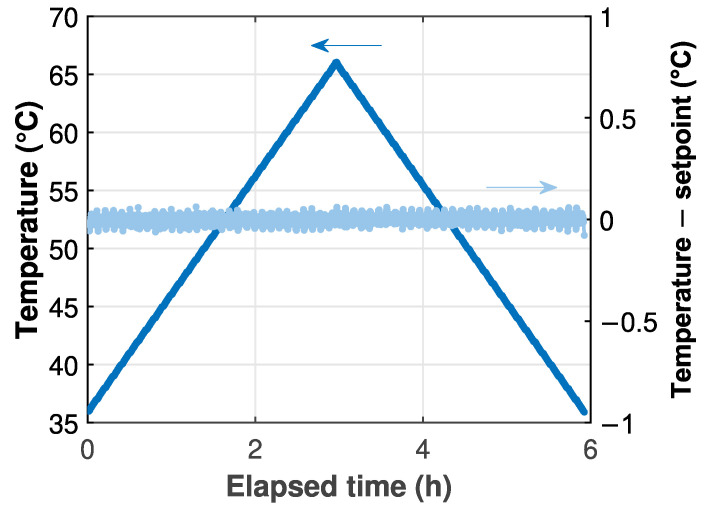
Measured temperature (**left axis**) and difference with the setpoint (**right**) as function of the time since the start of the measurement of Figure 4.

**Figure A4:**
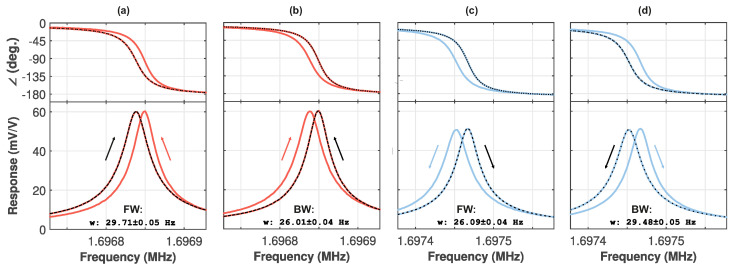
NWA traces at 50 °C for increasing temperature (**a**,**b**) [red] and while cooling (**c**,**d**) [light blue] with fits [black]. The data in (**a**,**b**) and in (**c**,**d**) is identical, but either the forward (**a**,**c**) or the backward (**b**,**d**) direction (arrows) of the NWA trace is fitted with a harmonic oscillator response (black). The resulting linewidth *w* is indicated. All NWA traces have a span of 200 Hz and 101 points per sweep direction (202 in total).

**Figure A6:**
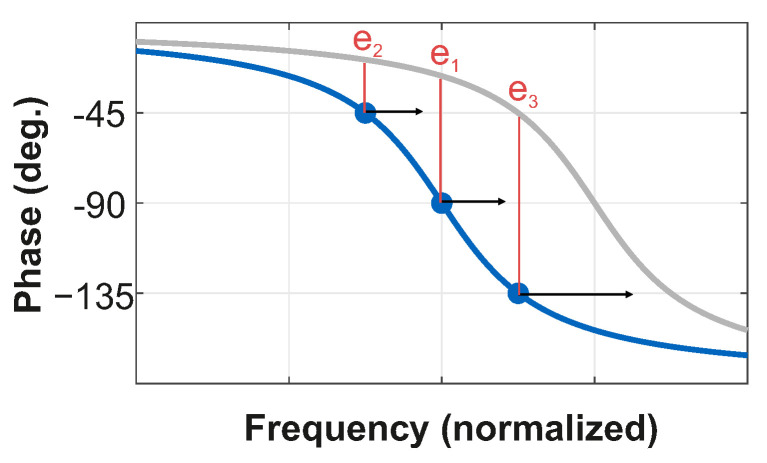
Illustration of the errors (indicated in red) of the three PLLs during a frequency shift from the original (blue) to the shifted position (gray). The resulting, unequal increments by the three PLLs are indicated in black.

**Figure A7:**
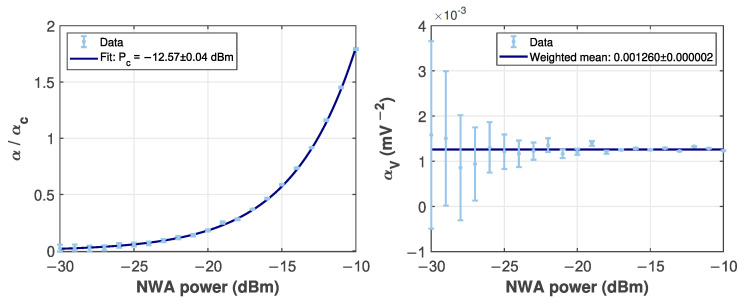
Normalized Duffing parameter (**left**) as determined by fitting the phase of the driven responses [26] vs. driving power $P_{\mathrm{drive}}$. A subset of these responses was shown in Figure 8a. The solid line is the fitted expected linear (quadratic) dependence on the pump power (amplitude). (**right**) shows $\alpha_{V}$ and its fit uncertainty, together with the weighted mean value.

## Data Availability

The data that support the findings of this study are available from the corresponding author upon reasonable request.

## Abbreviations

The following abbreviations are used in this work:

PLL	Phase-Locked Loop
NWA	Network Analyzer
RTK	Real Time Kit
MEMS	Micro-Electro-Mechanical System
NEMS	Nano-Electro-Mechanical System
SiN	Silicon Nitride
FB	Feedback Signal

## Appendix A. Experimental Methods

Membranes similar to those in Ref. [37] were made as described by Hoch et al. [22] by lithographically defining and dry-etching release holes in 330-nm-thick high-stress SiN, followed by an isotropic wet etch of the now-exposed silicon oxide using buffered hydrofluoric acid. A 130 min release etch results in the formation of a fully suspended SiN membrane above a flat Si surface [22,37]. The oxide etch also removes part of the SiN, making the membrane thinner than the original thickness of the SiN thin film [38,39]. Unlike our previous studies with rectangular membranes and a square lattice [39], this membrane has a triangular lattice of release holes and a hexagonal outline [26].

We use our interferometry setup [Figure 2], which is detailed in our previous work [22,23,39] to measure the vibrations of the membrane in vacuum. In summary, a HeNe laser is focused on the membrane, and the probing location is set with a stepper-motor-driven xy-translation stage. The sample is mounted on top of a Peltier element to control the temperature, which in turn heats the entire chip. Except for the temperature-ramp measurement, the temperature is kept stable at 36 °C. When the membrane moves, the reflectivity changes and is recorded by the photodetector (interferometric readout). A bias-tee splits the electrical signal from the photo detector into a d.c. part, which is used for the reflectivity measurement, and an a.c. part containing the vibration signal. Vibrations are excited with a piezoelectric element underneath the sample, which is driven by the output of the lock-in amplifier (Zurich Instruments HF2). This lock-in can also be used to record frequency sweeps, as an NWA would. We use a sweep over a large frequency range to determine the resonance frequency [Figure 3a].

Experimentally, the measured response is shifted relative to ∠H due to electrical delays and the excitation with the piezo. This device- and setup-dependent shift is accounted for in the actual values used for the PLLs in our experiments. From an initial frequency sweep, we find about $-90^{\circ }$. For clarity, we always give phases with this shift subtracted, i.e., the nominal setpoints of the three PLLs are $-90^{\circ }$, $-45^{\circ }$, and $-135^{\circ }$, respectively. Also, the offset is subtracted from the data presented in this work [e.g., in Figure 3c] so that it directly compares to the theoretical phase response.

As in our previous works on efficient mode mapping [22,23], up to six PLLs (here, only three are used) are implemented digitally in LabVIEW by processing the signals from the lockin and setting its output frequencies (which equal the demodulation frequencies) using a proportional-integral control loop. Specifically, the PLL program runs independently from the main measurement program. The former communicates with the lock-in amplifier, whereas the latter controls all other measurement equipment. The two LabVIEW programs communicate via global variables. The PLL program loops over all active PLLs; the actual code is implemented manually in a sub-VI. The PLL program is free-running, and a dwell time of 0.1 s is used to reduce the update rate to about 7 Hz. This is slightly faster than the largest PLL-demodulation bandwidth of the lock-in (4 Hz) employed throughout our investigation. Care is taken to ensure the demodulator bandwidth is narrow enough that nearby frequencies are not picked up significantly. Typically, a bandwidth of 1 Hz was used. Cross-talk [23] is not significant in the measurements presented here. Post-processing of the measurement data is done using Matlab.

For the measurements in Figure 6, a feedback loop is added to the setup, as schematically shown in Figure A1. The feedback is implemented using the digital-signal processor of the lock-in [29,40], namely the Real Time Kit (RTK). It updates at 14,400 Sa/s and has a bandwidth of 5000 Hz around 1,692,550 Hz. The lock-in outputs the feedback signal (FB) as well as the drive for all three PLLs. This is forwarded to the piezo, which actuates the mechanical resonator with an inertial force *F*, and read out via reflected light. The gain $g_{\mathrm{FB}}$ is stated in V/V so that the total loop gain is given by the product of the (open-loop) driven response y(f) and the feedback gain, including the feedback phase shift $\theta_{\mathrm{FB}}$. Like the PLL setpoints, the stated feedback phase is corrected for the overall shift so that $\theta_{\mathrm{FB}}=-90{}^{\circ}$ corresponds to an increased damping rate [28]—at least for a positive transduction—as can be seen in Figure A2. See Ref. [29] for details on a similar implementation of feedback using the lockin amplifier.

## Appendix B. Temperature Ramp Measurement

Figure A3 shows the evolution of the measured temperature of the measurement shown in Figure 4 of the main text. The thermometer (Pt-1000) is located inside the mounting plate for the PCB with the piezo and chip. This aluminum plate sits on top of a Peltier heater, which is controlled via the PC. The measured temperature follows the setpoint of the temperature control closely, but the sample temperature may be lagging behind due to the thermal resistance of the PCB and piezo on which it is glued, causing the hysteresis visible in Figure 4a.

The temperature ramp of ∼±10 K/h, in combination with a temperature coefficient ∼+3 kHz/K and a linewidth w∼22 Hz, means that during the ∼33 s that it takes to measure a network trace, the resonance shifts significantly, as shown in Figure A4. Also, the apparent resonance widens or narrows, depending on whether the sweep direction and frequency shift direction coincide, or not. Without considering this, the linewidth extracted from NWA measurements would thus not be correct. Hence, for the NWA data in Figure 4, the average value of the fit parameters in the forward and backward driven response frequency sweep are used. Moreover, the large frequency shift of almost 10 kHz means that either the span has to at least as large, which—in combination with the narrow linewidth—implies a very long measurement, or the center frequency has to be well chosen. For every NWA calibration (done every 100 measurements, roughly every 6 min.), the latest value of $f_{1}$ was used for the center of the NWA span. In other words, the PLL have to support the NWA measurements.

Finally, we note that the temperature sweep was measured after an improvement to the setup, which resulted in larger absolute signals and a different phase offset than the other measurements presented in this work.

## Appendix C. Map Without Feedback

Figure A5 shows mapping measurement as in Figure 6 of the main text, but now without feedback. The slightly thicker SiN (Our working hypothesis is that this is because at that location the SiN is protected against the oxide etchant for some time, due to an air bubble or organic residue.) on the upper right corner gives a different sign of the transduction [31,41]. This is also visible as discolorations in the micrograph shown in panel Figure A5f—both in the supported (brown → purple) and in the suspended (light blue → yellow) parts. The light gray area separating the blue and red regions in the mode map (b) coincides with the minimum in the reflectivity map (a). This is caused by a SiN-thickness-change-induced shift of reflectivity-versus-displacement-fringe R(u) from the side with positive dR/du, through a minimum, to the side with negative transduction [26,31] as illustrated in Figure A5e: The detected signal (Figure A1) can be described as:

(A1) $$V_{\det}=\frac{\partial V_{\det}}{\partial P_{\det}}\;P_{L}\;R\left(u\right),$$

with $P_{\det}$ the power of the reflected laser light impinging on the photodetector, $P_{L}$, the laser power, and R(u) the displacement-dependent reflectivity. The transduction is then given by:

(A2) $$\frac{\partial V_{\det}}{\partial u}=\frac{\partial R(u)}{\partial u}\frac{\partial V_{\det}}{\partial P_{\det}}\;P_{L}.$$

As a function of displacement, everything included in the readout, $\frac{\partial V_{\det}}{\partial P_{\det}}$—as well as the laser power $P_{L}$—remains constant. Therefore, only the changes in transduction depend on changes in reflectivity with respect to displacement. The schematic in Figure A5e shows this effect. Every sinusoidal curve R(u) depicts a different SiN thickness. The three dots indicate three different thickness regions at the same displacement. At each point, the reflectivity signal has a different slope (dotted lines): either positive (red), zero (gray) or negative (blue). Each location would have a different sign in the transduction which modulates the mode map in (b). This explains the sign reversal, despite the fundamental mode not having a nodal line [31]. Demonstrating the operation of our multi-tone PLL method in the presence of this and other features shows the robustness in real-world applications.

Without the feedback, the (estimated) linewidth map in (d) is constant over the membrane. The frequency shift $\Delta f_{0}=f_{1}\left(x,y\right)-\langle f_{1}\rangle$ shows a slow drift of a few Hz (green → yellow) as well as a number of localized spots where the frequency drops sharply. These coincide with regions of low reflectivity *R* and signal *S*, supporting that these are dirt particles/debris that absorb part of the laser light, thereby heating the membrane locally, lowering its frequency [27,42]. Near those locations, *w* remains constant and there is no estimate available exactly at the particle position (white). The particles are not very well visible in the micrograph; only the two spots at the bottom can (barely) be seen and are indicated by the black arrows.

## Appendix D. Details on the PLL

### Appendix D.1. PLL Asymmetry

The concept behind the PLL tracking is a PI(D) control. Here, only the proportional (‘P’) and integral (‘I’) terms of the PID are used. For tracking, we lock onto the phase response, and the PLL aims to maintain a stable phase value, taking past errors $e_{j\leq i}$ into account (‘I’ term). Suppose that initially the PLL is locked. If the frequency now starts shifting, e.g., due to a change in temperature, the PLL will try to adjust the frequency accordingly. The amount of P-shift is determined from the previous error $e_{i}$. When tracking three frequencies simultaneously, the error varies across the PLLs due to the shape of the phase response; see Figure A6, indicated in red. Taking the center-PLL ($-90^{\circ }$) as a reference, the second one at $-45^{\circ }$ has less error in the same amount of time, whereas the third one at $-135^{\circ }$ has more than the center, leading to different frequency adjustments for each setpoint by the ’P’ term. When the frequency shifts continuously, this situation persists, and the ’I’ term also contributes. Also, this one is the smallest for the smaller errors of the second ($-45^{\circ }$) PLL, at least until the PLLs almost “catch up” and the errors decrease. Thus, an asymmetry arises between the second and third frequency, as was observed in Figure 3a.

### Appendix D.2. PLL Method vs. NWA Measurements

In order to compare the speed of the two measurement approaches, first, the parameters of both are discussed. The speed of the PLLs is influenced by the update rate (150ms), the P and I parameters, and the locking amplifier. The lock-in has a sample rate of 225samples/s and a bandwidth of 1Hz. The latter is the slowest element of the PLL.

The NWA speed is defined by the resolution bandwidth and the number of points per sweep. One can increase the resolution bandwidth and decrease the number of points to increase measurement speed. But after a certain point, the resonator can not keep up with the sweep, and the shape of the resonance will be deformed. This complicates the postprocessing, as a model must be developed to fit the resulting traces. Additionally, e.g., when following a temperature sweep, the frequency range must be large enough to capture the shifting resonance. This adds a lot of low-response data that the PLLs do not have to cover, as it tracks the frequency, being therefore fundamentally faster.

To give an example for our specific case, the resolution of the PLL maps is 121 × 145 points. The time per point is about 2s. Spanning over the whole membrane, the measurement time is ≈12 h. This is, however, mainly limited by stage travel and other measurement overhead, and not by the PLLs themselves. One NWA trace takes 33 s, so the whole map would take ≈7 days. In our case, we find that the PLL method is about 14 times faster than taking an NWA map.

## Appendix E. Nonlinear Regime

Looking at Figure A1 shows that the driven response is given by:

(A3) $$y\left(f\right)\equiv \frac{V_{\det}\left(f\right)}{V_{\mathrm{drive}}\left(f\right)}=A_{\mathrm{piezo}}H\left(f\right)\frac{dV_{\det}}{du}.$$

This holds for both the linear and nonlinear regime; the difference lies in the resonator response H(f), which is either a Lorentzian (cf. Equation (1)) or is a Duffing response. We use the following fit function for the driven response y(f) [26]:

(A4) $$y\left(f\right)=\frac{y_{\max}w/2}{f_{0}(1+\frac{3}{8}\alpha_{y}{\left|y\right|}^{2})-f+iw/2}e^{i\psi -2\pi i(f-f_{0})\tau }.$$

The fit parameters are $f_{0}$, *w*, $y_{\max}$, $\alpha_{y}$, ψ and τ. Note the presence of ${\left|y\right|}^{2}$ in the denominator and that for $\alpha_{y}=0$, Equation (A4) reduces to a Lorentzian. The parameters $f_{0}$, *w*, $y_{\max}$ are the resonance frequency, linewidth and maximum response in the linear regime, respectively. ψ takes the any additional phase at $f_{0}$ into account and τ a (group) delay. $\alpha_{y}$ quantifies the nonlinearity and has units of ${\left(V/V\right)}^{-2}$. Its critical value $\alpha_{c}$ is the lowest value of $|\alpha_{y}|$ where |y| just becomes vertical [43]; beyond that the response becomes hysteretic. In the Lorentzian approximation, its value is $\alpha_{c}=\frac{32}{27}\sqrt{3}w/\left(f_{0}y_{\max}^{2}\right)$ [44]. Hence, once the fit parameters *w*, $f_{0}$ and $y_{\max}$ are determined, the value of $\alpha_{c}$ is fixed. Note that $\alpha_{c}$ is independent of the driving strength. Still, $\alpha_{y}$ depends linearly on the driving power $P_{\mathrm{drive}}\propto V_{\mathrm{drive}}^{2}$. Note that in our experiments, there is a stiffening nonlinearity (i.e., $\alpha_{y}\geq 0$), so this can be written as: $\alpha_{y}=\alpha_{c}P_{\mathrm{drive}}/P_{c}=\alpha_{c}{\left(V_{\mathrm{drive}}/V_{c}\right)}^{2}$, where the latter expression uses the drive voltage amplitude $V_{\mathrm{drive}}\left(f\right)$.

Figure A7 shows the behavior of the fitted normalized Duffing parameter for increasing driving power. The power is varied from $P_{\mathrm{drive}}=-30\;dBm$ to −10 dBm and at every integer value of the drive power, a NWA trace is recorded with the lock-in. As the response at high power is influenced by a nonlinear readout, the phase ∠y(f) is used for fitting followed by determining $y_{\max}$ from the linear part as detailed in Ref. [26]. From the dependence of the normalized Duffing parameter on the driving strength, the critical driving power $P_{c}$—where the normalized Duffing parameter equals 1, i.e., $\alpha_{y}\left(P_{c}\right)=\alpha_{c}$—is found at $P_{c}=-12.57\;dBm$.

For the discussion of the pump-probe experiments in Figure 8 of the main text, Equation (A4) is rewritten from normalized responses y(f)—as measured by e.g., an NWA—to actual voltages: $V_{\det},\mathrm{pump}\left(f\right)=y\left(f\right)V_{\mathrm{drive}},\mathrm{pump}\left(f\right)$. For simplicity, we will assume that ψ=τ=0. Also, it is assumed that the readout is not nonlinear. Then, by expanding the underlying equations of motion for a weak probe tone at frequency ν around the steady-state solution for the strong pump, the probe response is found [35,36]. In the following, we use uppercase *V*s for the pump and lowercase *v*s for the weak probe. Their frequencies are labeled *f* and ν, respectively. Combining all of this, gives the following:

(A5) $$\begin{array}{ccc} V_{\det},\mathrm{pump}\left(f\right) & = & \frac{V_{\max}w/2}{f_{0}(1+\;\frac{3}{8}\alpha_{V}|V_{\det},\mathrm{pump}{|}^{2})-f+\frac{iw}{2}} \end{array}$$

(A6) $$\begin{array}{ccc} v_{\det},\mathrm{probe}\left(\nu \right) & = & \frac{v_{\max}w/2}{f_{0}(1+2\frac{3}{8}\alpha_{V}|V_{\det},\mathrm{pump}{|}^{2})-\nu +\frac{iw}{2}-\frac{(\frac{3}{8}\alpha_{V}|V_{\det},\mathrm{pump}{{|}^{2})}^{2}}{f_{0}(1+2\frac{3}{8}\alpha_{V}|V_{\det},\mathrm{pump}{|}^{2})-\left(2f-\nu \right)-\frac{iw}{2}}}. \end{array}$$

Here, $V_{\max}=y_{\max}V_{\mathrm{drive}},\mathrm{pump}$ and $v_{\max}=y_{\max}v_{\mathrm{drive}},\mathrm{probe}$. Similarly, $\alpha_{V}=\alpha_{y}{\left|V_{\mathrm{drive}},\mathrm{pump}\right|}^{-2}$ is the Duffing parameter in per-Volt-squared, which is independent of the pump strength as $\alpha_{y}\propto V_{\mathrm{drive}}^{2}$ as discussed above. This is verified in Figure A7.

The equation for the pump [Equation (A5)] is just a rewritten version of Equation (A4), and it shows that the detector signal at the pump frequency solely depends on the pump, and not on the weak probe. Now, when using a PLL (“1”) to lock the pump to $\phi_{1}=-90^{\circ }$ as is done in the experiments, its frequency ($f\to f_{1}$) will track the “backbone curve” [21] $f_{0}(1\;+\;\frac{3}{8}\alpha_{V}|V_{\max}{|}^{2})$ when changing the pump strength (i.e., $V_{\mathrm{drive,pump}}\propto V_{\max}$). This is the straight blue line in Figure 8c.

The expression for the probe signal [Equation (A6)] is more complicated than that for the pump, but it is noted that the denominator does not contain probe voltages *v*, only pump amplitudes *V*. The former only appears (as $v_{\max}\propto v_{\mathrm{drive,probe}}$) in the numerator and $v_{\mathrm{det,probe}}\left(\nu \right)$ is linear in $v_{\mathrm{drive,probe}}$ [36]. This linearity also indicates that multiple weak probes (i.e., PLLs 2 and 3) will not influence each other. We define the probe response (in V/V) as $p\left(\nu \right)\equiv v_{\mathrm{det,probe}}\left(\nu \right)/v_{\mathrm{drive,probe}}\left(\nu \right)$. Such measured probe responses were shown in Figure 8b, whereas a colormap of the phase ∠p(ν) calculated with Equation (A6) vs. probe frequency and the normalized Duffing parameter was shown in Figure 8c. The PLLs of the weak probes would follow the contour lines (black) corresponding to their setpoints. It is noted that setpoints more negative than the pump are shifted to the right, but never cross, whereas PLLs with less negative setpoints may have to cross the pump frequency. Experimentally, this will give issues with the PLL operation. Likewise, when increasing or decreasing the pump strength, a nonmonotonous contour line would require the PLL to make sudden jumps, which is undesirable.

Finally, the peak shape of the probe shows a complicated resonance due to the nature of Equation (A6). The first part of its denominator indicates that the probe probes a Lorentzian resonance at $\nu =f_{0}(1+2\frac{3}{8}\alpha_{V}|V_{\mathrm{det,pump}}{|}^{2})$. Its shift is thus twice that of the pump [35], and this resonance is intimately related to the libration eigenvalues [45]. In principle, it could be tracked by a probe locked to $-90^{\circ }$. The second part of the denominator is a fraction that is caused by four-wave-mixing processes [36] and has a resonance when 2f−ν matches $f_{0}(1+2\frac{3}{8}\alpha_{V}|V_{\mathrm{det,pump}}{|}^{2})$. This term is responsible for the light blue region where ∠p>0 in Figure 8c. For a pump locked to $-90^{\circ }$, this term is largest at $\nu =f_{0}$.
